# Aquaglyceroporins but not orthodox aquaporins are involved in the cryotolerance of pig spermatozoa

**DOI:** 10.1186/s40104-019-0388-8

**Published:** 2019-10-14

**Authors:** Ariadna Delgado-Bermúdez, Marc Llavanera, Leira Fernández-Bastit, Sandra Recuero, Yentel Mateo-Otero, Sergi Bonet, Isabel Barranco, Beatriz Fernández-Fuertes, Marc Yeste

**Affiliations:** Biotechnology of Animal and Human Reproduction (TechnoSperm), Unit of Cell Biology, Department of Biology, Faculty of Sciences, Institute of Food and Agricultural Technology, University of Girona, C/Maria Aurèlia Campany, 69, Campus Montilivi, E-17003 Girona, Spain

**Keywords:** Acetazolamide, Aquaporins, Boar, Phloretin, Propanediol, Sperm

## Abstract

**Background:**

Aquaporins (AQPs) are a family of transmembrane water channels that includes orthodox AQPs, aquaglyceroporins (GLPs) and superAQPs. AQP3, AQP7, AQP9 and AQP11 have been identified in boar sperm, and they are crucial for sperm maturation and osmoregulation. Water exchange is an important event in cryopreservation, which is the most efficient method for long-term storage of sperm. However, the freeze-thaw process leads to sperm damage and a loss of fertilizing potential. Assuming that the quality of frozen-thawed sperm partially depends on the regulation of osmolality variations during this process, AQPs might play a crucial role in boar semen freezability. In this context, the aim of this study was to unravel the functional relevance of the different groups of AQPs for boar sperm cryotolerance through three different inhibitors.

**Results:**

Inhibition of different groups of AQPs was found to have different effects on boar sperm cryotolerance. Whereas the use of 1,3-propanediol (PDO), an inhibitor of orthodox AQPs and GLPs, decreased total motility (*P* < 0.05), it increased post-thaw sperm viability, lowered membrane lipid disorder and increased mitochondrial membrane potential (MMP) (*P* < 0.05). When acetazolamide (AC) was used as an inhibitor of orthodox AQPs, the effects on post-thaw sperm quality were restricted to a mild increase in MMP in the presence of the intermediate concentration at 30 min post-thaw and an increase in superoxide levels (*P* < 0.05). Finally, the addition of phloretin (PHL), a GLP inhibitor, had detrimental effects on post-thaw total and progressive sperm motilities, viability and lipid membrane disorder (*P* < 0.05).

**Conclusions:**

The effects of the different inhibitors suggest that GLPs rather than orthodox AQPs are relevant for boar sperm freezability. Moreover, the positive effect of PDO on sperm quality suggests a cryoprotective role for this molecule.

## Introduction

Cell function and survival are strictly related to metabolite concentration, which, in turn, depends on plasma membrane permeability to water and solutes. Considering plasma membrane hydrophobicity, mechanisms other than simple diffusion are required to allow water transport across the plasma membrane in certain cell functions [1]. Aquaporins (AQPs) are a family of ubiquitous integral transmembrane proteins that allow the passive transport of water through cell membranes. Moreover, some AQPs also facilitate the transport of small solutes, such as glycerol or hydrogen peroxide [2]. Mammalian AQPs are classified according to their sequence similarity and substrate affinity into orthodox AQPs, aquaglyceroporins (GLPs) and superaquaporins (superAQPs). Orthodox AQPs are permeable to water, and this group is formed by AQP0, AQP1, AQP2, AQP4, AQP5, AQP6 and AQP8. Concerning the group of GLPs, it comprises AQP3, AQP7, AQP9 and AQP10, which are permeable to water, but also to glycerol, urea and other small electrolytes. Finally, AQP11 and AQP12 belong to the superAQPs group, which are specifically localized in the membrane of intracellular organelles and regulate organelle volume and intravesicular homeostasis while being involved in both water and glycerol transport [2].

AQPs have been identified in mammalian sperm cells with differences between species. While AQP3, AQP7 and AQP11 have been identified in sperm from boar [3, 4], mouse [5–7], human [8, 9], cattle [10, 11] and stallion [12], AQP8 has only been observed in mouse [6] and human [8, 9] sperm, and AQP9 has been identified in boar sperm [13]. In mammalian sperm, AQPs play an important role in the adaptation to the osmotic variation caused by the exposure to the female reproductive tract, which is also involved in sperm motility activation upon ejaculation (reviewed in [2]). In addition, AQPs play a key role during spermatogenesis [14], since they contribute to cytoplasm reduction from spermatids to spermatozoa.

Cryopreservation is the most efficient method for the long-term storage of sperm. However, the freeze-thawing process damages spermatozoa in terms of plasma membrane destabilization, nuclear alterations, reduction of mitochondrial activity, changes in sperm proteins and a decrease in sperm motility [15, 16]. Cryoinjury is mainly inflicted during the processes of freezing and thawing, when extracellular water freezes and intracellular water is lost. In this context, the penetration of permeating cryoprotective agents (CPAs), such as glycerol, is essential to minimize cryoinjuries [15, 17, 18]. The resilience of sperm cells to cryopreservation is also known as freezability or cryotolerance, which differs between species according to differences in membrane composition [15, 17, 18]. Among the AQPs identified in boar sperm cells, AQP3 and AQP7 are associated to sperm cryotolerance, which suggests that they could be used as freezability biomarkers [19].

GLPs substrate affinity and their role in volume regulation is the potential mechanism through which AQP3 and AQP7 appear to be related to sperm cryotolerance. Therefore, it is reasonable to suggest that the inhibition of sperm AQPs would impair their resilience to freeze-thawing procedures. Different AQP inhibitors have previously been tested in other cell types, such as 1,3-propanediol (PDO), acetazolamide (AC) and phloretin (PHL). PDO occludes the pore channel of orthodox AQPs, such as AQP1, AQP2, AQP4 and AQP5 [20, 21] from the outer side of the plasma membrane, but it is also able to penetrate through *Plasmodium falciparum* PfAQP (which is an analogue of AQP3, AQP7 and AQP9) and to inhibit this GLP analogue from the inner part of the plasma membrane [21]. AC has inhibitory effects on the orthodox AQPs, AQP1 and AQP4 [22, 23], whereas PHL inhibits AQP3 and AQP9, which are both GLPs [24, 25]. Against this background, this study aims to elucidate the functional relevance of orthodox AQPs and GLPs during boar sperm cryopreservation, through their inhibition by the aforementioned agents.

## Methods

### Boars and ejaculates

A total of 20 ejaculates from separate Piétrain boars (*n* = 20) were used in this study. The boars were housed in a local farm (Semen Cardona, Cardona, Barcelona, Spain) with controlled climatic conditions, and were fed a standard diet. Sperm-rich fractions were collected manually, and subsequently diluted 1:1 (*v*:*v*) in a commercial semen extender (Vitasem LD; Magapor S.L., Zaragoza, Spain). After collection, ejaculates were stored in bags at 17 °C and transported to the laboratory within 5 h after extraction. Once in the laboratory, the ejaculates were pooled in pairs, and each pool was split into two different fractions. The first one was used to evaluate the quality parameters in fresh semen; the second one was divided into seven different sub-fractions, which were cryopreserved with or without different concentrations of the three AQP inhibitors.

### AQP inhibitors

Prior to cryopreservation, three AQP inhibitors were added to the semen samples: 1,3-propanediol (PDO, Sigma-Aldrich, St. Louis, MO, USA), acetazolamide (AC, Sigma-Aldrich), and phloretin (PHL, Sigma-Aldrich). PDO was diluted in cryopreservation medium (LEYGO medium, see composition in the following section) to a working concentration of 100 mmol/L, AC was diluted in dimethyl sulfoxide (DMSO, Sigma-Aldrich) to a working concentration of 450 mmol/L, and PHL was diluted in methanol (Fisher Chemical, ThermoFisher Scientific; Waltham, Massachusetts, USA) to a working concentration of 365 mmol/L. For each inhibitor, three different concentrations were tested: 0.1, 1 and 10 mmol/L for PDO; and 250, 500 and 1000 μmol/L for AC and PHL. It is worth mentioning that in the case of treatments containing PDO and AC, samples were exposed to methanol or DMSO at concentrations lower than 0.5% (*v/v*). These concentrations showed no detrimental effects on the sperm quality parameters (data not shown).

### Boar sperm cryopreservation

Cryopreservation of boar sperm was performed to determine whether the three AQP inhibitors affected their cryotolerance. The fraction of the pool intended for cryopreservation was divided into 50-mL aliquots and centrifuged at 15 °C and 2400×*g* for 3 min. After discarding the supernatants, pellets were resuspended to a final concentration of 1.5 × 10^9^ spermatozoa/mL in β-lactose-egg yolk freezing medium (LEY), which consisted of 80% (*v/v*) lactose (Sigma-Aldrich) and 20% (*v/v*) egg yolk. Subsequently, sperm were cooled down to 5 °C with a cooling ramp of − 0.1 °C/min (180 min) in a programmable, controlled-rate freezer (Icecube14S-B; Minitüb Ibérica SL; Tarragona, Spain). Sperm were then diluted to a final concentration of 1 × 10^9^ spermatozoa/mL in LEYGO medium, which consisted of LEY medium supplemented with 6% (*v*/*v*) glycerol (Sigma-Aldrich) and 1.5% Orvus ES Paste (Equex STM; Nova Chemical Sales Inc., Scituate, MA, USA). At this point, the suspension was divided into seven sub-fractions: one for each concentration and inhibitor, and a non-treated control. Samples were packed into distinct 0.5 mL plastic straws (Minitüb Ibérica, S.L.). Afterwards, straws were placed in a controlled-rate, programmable freezer (Icetube 14S-B; Minitub Ibérica, S.L), using the following cooling rates [26]: − 6 °C/min from 5 to − 5 °C (100 s); − 39.82 °C/min from − 5 to − 80 °C (113 s); hold at − 80 °C for 30 s; and cooled at − 60 °C/min from − 80 to − 150 °C (70 s). Finally, straws were plunged into liquid nitrogen (− 196 °C) for storage.

Analysis of sperm quality parameters was performed after thawing. With this purpose, two straws per treatment and pool were immersed and agitated in a water bath at 38 °C for 15 s. Thereafter, sperm samples were diluted 1:3 (*v*:*v*) in pre-warmed Beltsville Thawing Solution (BTS) [27]. Diluted, frozen-thawed samples were incubated at 38 °C for 240 min, and sperm quality was evaluated at two different time points: 30 min and 240 min.

### Sperm motility

Sperm motility was evaluated in both fresh and frozen-thawed semen samples through a computer-assisted sperm analysis (CASA) system, which consisted of a phase contrast microscope (Olympus BX41; Olympus, Tokyo, Japan) equipped with a video camera and ISAS software (Integrated Sperm Analysis System V1.0; Proiser SL, Valencia, Spain). Extended samples were incubated at 38 °C for 15 min before sperm motility assessment, whereas frozen-thawed samples were directly examined after 30 min and 240 min of incubation. Following incubation, 5 μL of the sperm suspension were placed onto a pre-warmed Makler counting chamber (Sefi-Medical Instruments, Haifa, Israel) and observed under a negative phase-contrast field (Olympus 10× 0.30 PLAN objective; Olympus). At least 1000 sperm were examined per replicate, and three replicates were evaluated per sample.

In each motility assessment, the following parameters were recorded: total (TMOT, %) and progressive sperm motility (PMOT, %); curvilinear velocity (VCL, μm/s); straight line velocity (VSL, μm/s); average path velocity (VAP, μm/s); amplitude of lateral head displacement (ALH, μm); beat cross frequency (BCF, Hz); linearity (LIN, %), which was calculated assuming that LIN=VSL/VCL × 100; straightness (STR, %), resulting from VSL/VAP × 100; and motility parameter wobble (WOB, %), obtained from VAP/VCL × 100. A sperm cell was considered to be motile when its VAP was equal to or higher than 10 μm/s and progressively motile when its STR was equal to or higher than 45%. The corresponding mean ± standard error of the mean (SEM) was calculated for each parameter.

### Flow cytometry

As mentioned above, five different parameters were assessed through flow cytometry in both fresh and frozen-thawed semen samples: viability, membrane lipid disorder, mitochondrial membrane potential (MMP), intracellular levels of superoxide (O_2_^−^•) radicals and intracellular levels of hydrogen peroxide (H_2_O_2_). All fluorochromes used were purchased from ThermoFisher Scientific. For proper staining, all samples were diluted to a final concentration of 1 × 10^6^ spermatozoa/mL, and after the addition of the corresponding fluorochromes, they were incubated at 38 °C in the dark. A total of three replicates per sample were assessed for each parameter.

Samples were evaluated using a Cell Laboratory QuantaSC™ cytometer (Beckman Coulter; Fullerton, CA, USA). All samples were excited with an argon ion laser (488 nm) set at a power of 22 mW. Cell diameter/volume was assessed employing the Coulter principle for volume assessment using the Cell Lab Quanta™ SC cytometer, which is based on measuring changes in electrical resistance produced in an electrolyte solution by suspended, non-conductive particles. In this system, forward scatter (FS) is replaced by electronic volume (EV). Furthermore, 10-μm Flow-Check fluorospheres (Beckman Coulter) were used for EV-channel calibration, by positioning this size of bead at channel 200 on the EV-scale.

Three optical filters were used: FL1 (Dichroic/Splitter, DRLP: 550 nm, BP filter: 525 nm, detection width: 505–545 nm), FL2 (DRLP: 600 nm, BP filter: 575 nm, detection width: 560–590 nm) and FL3 (LP filter: 670 nm/730 nm, detection width: 655–685 nm). The first one was used to detect green fluorescence from SYBR-14, YO-PRO-1, JC-1 monomers (JC-1_mon_) and 2′,7′-dichlorofluorescein (DCF^+^); the second one was used to detect orange fluorescence from JC-1 aggregates (JC-1_agg_); and the third one was used to detect red fluorescence from propidium iodide (PI), merocyanine 540 (M540) and ethidium (E^+^). The signal was logarithmically amplified, and the adjustment of photomultiplier settings was performed according to particular staining methods.

For all particles, EV and side scatter (SS) were measured and linearly recorded. The sheath flow rate was set at 4.17 μL/min and a minimum of 10,000 events were evaluated per replicate. On the EV channel, the analyzer threshold was adjusted to exclude subcellular debris (particle diameter < 7 μm) and cell aggregates (particle diameter > 12 μm). Therefore, on the basis of EV and SS distributions, the sperm-specific events were positively gated, whereas the others were gated out.

Subsequent data analysis was performed using Flowing Software (Ver. 2.5.1; University of Turku, Finland) and according to the recommendations of the International Society for Advancement of Cytometry (ISAC). The corresponding mean ± SEM was calculated for each parameter.

#### Plasma membrane integrity

Sperm viability was evaluated by the assessment of membrane integrity using the LIVE/DEAD Sperm Viability Kit (Molecular Probes, Eugene, OR, USA), following the protocol of Garner and Johnson [28]. In brief, sperm were incubated with SYBR-14 (final concentration: 100 nmol/L) for 10 min, PI was subsequently added (final concentration of 12 μmol/L) and sperm were incubated for an additional 5 min. In flow cytometry dot plots, three sperm populations were observed: 1) viable, green-stained sperm (SYBR-14^+^/PI^−^); 2) non-viable, red-stained sperm (SYBR-14^−^/PI^+^); 3) non-viable, both red- and green-stained sperm (SYBR-14^+^/PI^+^). The fourth dot population corresponded to unstained, non-sperm particles (SYBR-14^−^/PI^−^). Viable green-stained sperm were used to assess sperm viability, and SYBR-14 fluorescence spill over into FL3-channel was compensated (2.45%).

#### Sperm membrane lipid disorder

The evaluation of membrane lipid disorder was performed with M540 and YO-PRO-1 fluorochromes following a modification of the protocol from Rathi et al. [29] with minor modifications by Yeste et al. [30]. M540 detects a decrease in packing order of phospholipids in the outer monolayer of the plasma membrane. Sperm were incubated with M540 at a final concentration of 2.6 μmol/L, and with YO-PRO-1 at a final concentration of 25 nmol/L for 10 min. Four populations were identified in flow cytometry dot plots: 1) non-viable sperm with low membrane lipid disorder (M540^−^/YO-PRO-1^+^); 2) non-viable sperm with high membrane lipid disorder (M540^+^/YO-PRO-1^+^); 3) viable sperm with low membrane lipid disorder (M540^−^/YO-PRO-1^−^); and 4) viable sperm with high membrane lipid disorder (M540^+^/YO-PRO-1^−^). The percentage of viable sperm with low membrane lipid disorder (M540^−^/YO-PRO-1^−^) was corrected using the non-sperm particles from the SYBR-14/PI co-staining. Data were not compensated.

#### Mitochondrial membrane potential (MMP)

Determination of mitochondrial membrane potential (MMP) was performed with JC-1 following the procedure of Ortega-Ferrusola et al. [31] with minor modifications. Sperm were incubated with 0.3 μmol/L JC-1 for 30 min. Whereas high MMP causes JC-1 aggregate (JC-1_agg_) formation, in sperm cells with low MMP, JC-1 remains as a monomer (JC-1_mon_). Particles showing no fluorescence (either green or orange) were gated out. Flow cytometry dot plots allowed the identification of two different sperm populations: 1) sperm with low MMP (JC-1_mon_); and 2) sperm with high MMP (JC-1_agg_). Data were compensated, as green fluorescence from FL1-channel was subtracted from FL2-channel (51.70%).

#### Intracellular levels of superoxide (O_2_^−^•)

Intracellular levels of superoxide (O_2_^−^•) radicals were determined with hydroethidine (HE) and YO-PRO-1 fluorochromes following a modification of the procedure from Guthrie and Welch [32]. Sperm were incubated with 4 μmol/L HE and 40 nmol/L YO-PRO-1 for 20 min. HE permeates the sperm plasma membrane and is oxidized by O_2_^−^• to ethidium (E^+^) among other products. Flow cytometry dot plots allowed the identification of four different sperm populations: 1) non-viable sperm with low superoxide levels (E^−^/YO-PRO-1^+^); 2) non-viable sperm with high superoxide levels (E^+^/YO-PRO-1^+^); 3) viable sperm with low superoxide levels (E^−^/YO-PRO-1^−^); and 4) viable sperm with high superoxide levels (E^+^/YO-PRO-1^−^). The percentage of viable sperm with low superoxide levels (E^−^/YO-PRO-1^−^) was corrected using the non-sperm particles from the SYBR-14/PI co-staining. The percentages of sperm in the other three populations were recalculated and YO-PRO-1 spill over into the FL3-channel was compensated (5.06%).

#### Intracellular levels of hydrogen peroxide (H_2_O_2_)

Intracellular levels of hydrogen peroxide (H_2_O_2_) were determined with 2′,7′-dichlorodihydrofluorescein diacetate (H_2_DCFDA) and PI fluorochromes, following the procedure from Guthrie and Welch [32] with minor modifications. Sperm were incubated with 200 μmol/L H_2_DCFDA and 12 μmol/L PI for 30 min. H_2_DCFDA is a non-fluorescent probe that, after penetrating the sperm cell membrane, is intracellularly de-esterified and converted into highly fluorescent, 2′,7′-dichlorofluorescein (DCF^+^) upon oxidation. Flow cytometry dot plots allowed the identification of four different sperm cell populations: 1) viable sperm with high peroxide levels (DCF^+^/PI^−^); 2) non-viable sperm with high peroxide levels (DCF^+^/PI^+^); 3) viable sperm with low peroxide levels (DCF^−^/PI^−^); and 4) non-viable sperm with low peroxide levels (DCF^−^/PI ^+^). DCF^+^-spill over into FL3-channel was compensated (2.45%). The percentage of viable sperm with high peroxide levels (DCF^+^/PI^−^) was corrected using the non-sperm particles from the SYBR-14/PI co-staining.

### Statistical analyses

Data were analyzed using a statistical package (IBM SPSS Statistics 25.0; Armonk, New York, USA). Distribution of data and homogeneity of variances were tested through Shapiro-Wilk and Levene tests, respectively. A mixed model was subsequently run for each sperm parameter. The intra-subjects factor was the cryopreservation step (i.e. fresh, frozen-thawed at 30 min, frozen-thawed at 240 min), the fixed-effects factor (inter-subjects) was the treatment (C, and different concentrations of PDO, AC or PHL), and the random-effects factor was the ejaculate pool. A post-hoc Sidak test was used for pair-wise comparisons and the level of significance was set at *P* ≤ 0.05. Data are shown as mean ± SEM.

## Results

**Fig. 1:**
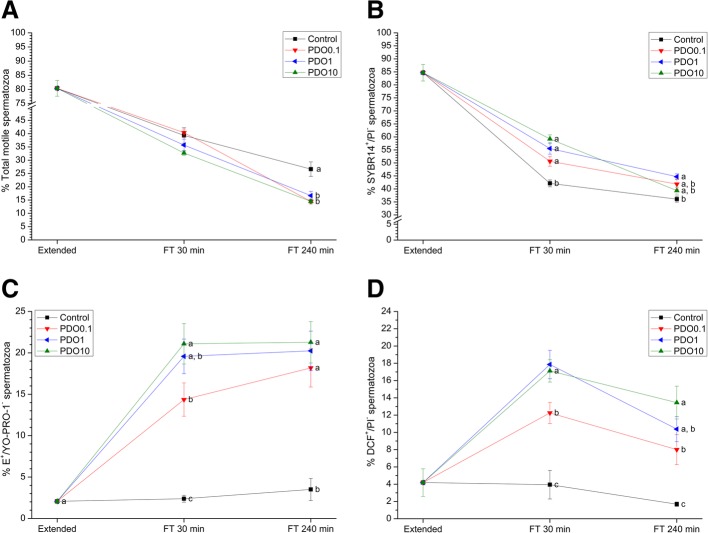
Sperm quality parameters in the presence of 1,3-propanediol (PDO) at three different concentrations (0.1 mmol/L, 1 mmol/L and 10 mmol/L) compared to samples exposed to extender alone. **a** Percentage of total motile spermatozoa (TMOT); **b** Percentage of viable sperm (SYBR-14^+^/PI^−^); **c** Superoxide levels (percentage of spermatozoa E^+^/YO-PRO-1^−^); **d** Peroxide levels (percentage of spermatozoa DCF^+^/PI^−^). Data were collected from fresh (extended) and frozen-thawed (FT) samples 30 and 240 min after thawing. Data reported as mean ± SEM. Different letters (**a-c**) indicate significant differences (*P* < 0.05) between treatments within a given time point

**Fig. 2:**
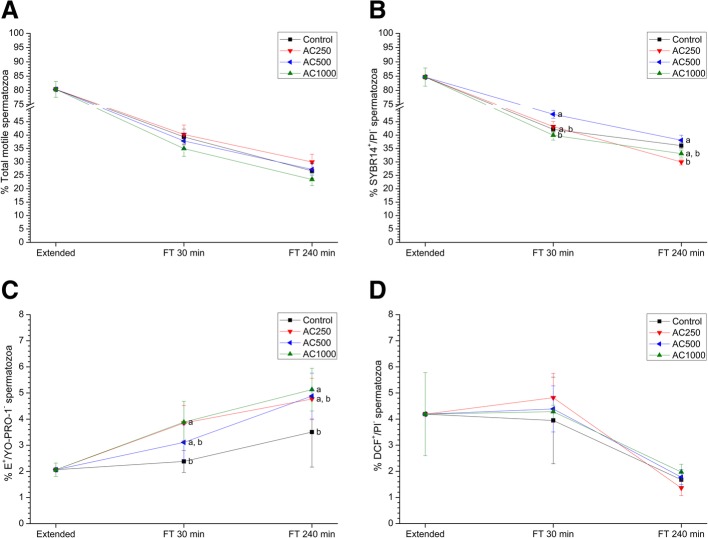
Sperm quality parameters in the presence of acetazolamide (AC) at three different concentrations (250 μmol/L, 500 μmol/L and 1000 μmol/L) compared to samples exposed to extender alone. **a** Percentage of total motile spermatozoa (TMOT); **b** Percentage of viable sperm (SYBR-14^+^/PI^−^); **c** Superoxide levels (percentage of spermatozoa E^+^/YO-PRO-1^−^); **d** Peroxide levels (percentage of spermatozoa DCF^+^/PI^−^). Data were collected from fresh (extended) and frozen-thawed (FT) samples 30 and 240 min after thawing. Data reported as mean ± SEM. Different letters (**a**, **b**) indicate significant differences (*P* < 0.05) between treatments within a given time point

**Fig. 3:**
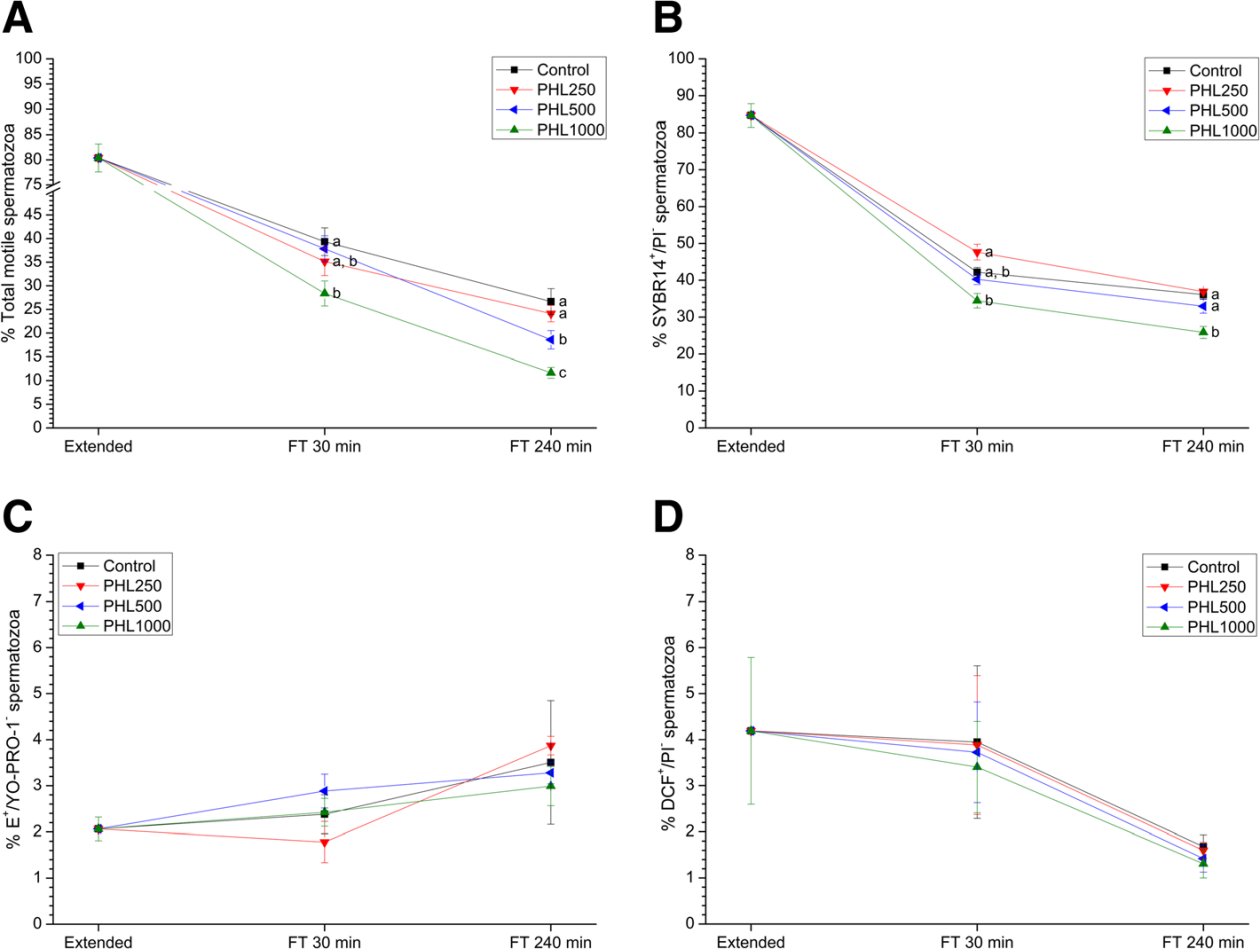
Sperm quality parameters in the presence of phloretin (PHL) at three different concentrations (250 μmol/L, 500 μmol/L and 1000 μmol/L) compared to samples exposed to extender alone. **a** Percentage of total motile spermatozoa (TMOT); **b** Percentage of viable sperm (SYBR-14^+^/PI^−^); **c** Superoxide levels (percentage of spermatozoa E^+^/YO-PRO-1^−^); **d** Peroxide levels (percentage of spermatozoa DCF^+^/PI^−^). Data were collected from fresh (extended) and frozen-thawed (FT) samples 30 and 240 min after thawing. Data reported as mean ± SEM. Different letters (**a-c**) indicate significant differences (*P* < 0.05) between treatments within a given time point

As mentioned above, sperm quality parameters were evaluated in both fresh and frozen-thawed samples to determine the effects of the AQP inhibition during cryopreservation. Because inhibitors were added immediately before cryopreservation, no effect of treatment was observed in fresh samples in any of the variables analyzed (*P* > 0.05; Figs. 1, 2, 3 and Tables 1, 2, 3).

**Table 1:** Sperm quality parameters from samples exposed to extender alone (control), or in the presence of 1,3-propanediol (PDO) at three different concentrations (0.1 mmol/L, 1 mmol/L and 10 mmol/L). The evaluated parameters were: percentage of progressively motile spermatozoa (PMOT), percentage of spermatozoa with low membrane lipid disorder (%M540^−^/YO-PRO-1^−^ sperm cells) and percentage of spermatozoa with high mitochondrial membrane potential (MMP; %JC1_agg_ spermatozoa). Determinations were performed in fresh (extended) and frozen-thawed (FT) spermatozoa after 30 and 240 min of thawing. Data reported as mean ± SEM

Variable	PDO concentration	Time point		
		Extended	FT-30 min	FT-240 min
%PMOT	CNT	69.87 ± 3.20	23.38 ± 2.74^a^	10.96 ± 1.12^a,b^
	0.1 mmol/L		26.62 ± 2.10^a^	7.60 ± 0.42^b^
	1 mmol/L		22.80 ± 2.12^a^	11.71 ± 1.07^a^
	10 mmol/L		24.94 ± 2.20^a^	8.48 ± 0.45^a,b^
%M540^−^/ YO-PRO-1^−^	CNT	80.83 ± 2.79	39.03 ± 1.49^a^	31.25 ± 1.06^a^
	0.1 mmol/L		48.12 ± 1.75^b^	37.56 ± 1.50^b,c^
	1 mmol/L		53.28 ± 2.10^b^	42.93 ± 1.38^c^
	10 mmol/L		56.02 ± 2.32^b^	36.50 ± 1.23^a,b^
%JC1_agg_	CNT	82.79 ± 2.41	31.95 ± 1.89^a^	26.01 ± 1.44^a^
	0.1 mmol/L		48.76 ± 1.85^b^	38.67 ± 1.58^b^
	1 mmol/L		40.95 ± 2.07^c^	34.39 ± 1.54^b, c^
	10 mmol/L		35.89 ± 2.06^a, c^	30.56 ± 1.29^a, c^

Different letters (^a–c^) indicate significant differences (*P* < 0.05) between treatments within a given time point for each parameter

**Table 2:** Sperm quality parameters from samples exposed to extender alone (control), or in the presence of acetazolamide (AC) at three different concentrations (250 μmol/L, 500 μmol/L and 1000 μmol/L). The evaluated parameters were: percentage of progressively motile spermatozoa (PMOT), percentage of spermatozoa with low membrane lipid disorder (%M540^−^/YO-PRO-1^−^ sperm cells) and percentage of spermatozoa with high mitochondrial membrane potential (MMP; %JC1_agg_ spermatozoa). Determinations were performed in fresh (extended) and frozen-thawed (FT) spermatozoa after 30 and 240 min of thawing. Data reported as mean ± SEM

Variable	AC concentration	Time point		
		Extended	FT-30 min	FT-240 min
%PMOT	CNT	69.87 ± 3.20	23.38 ± 2.74^a^	10.96 ± 1.12^a, b^
	250 μmol/L		25.34 ± 2.83^a^	13.47 ± 2.19^a^
	500 μmol/L		22.99 ± 2.57^a^	12.94 ± 1.98^a, b^
	1000 μmol/L		20.77 ± 2.29^a^	8.93 ± 1.30^b^
%M540^−^/ YO-PRO-1^−^	CNT	80.83 ± 2.79	39.03 ± 1.49^a, b^	31.25 ± 1.06^a, b^
	250 μmol/L		40.26 ± 1.73^a, b^	27.01 ± 0.98^a^
	500 μmol/L		46.39 ± 1.30^a^	35.28 ± 1.71^b^
	1000 μmol/L		36.03 ± 1.58^b^	30.52 ± 1.18^a, b^
%JC1_agg_	CNT	82.79 ± 2.41	31.95 ± 1.89^a^	26.01 ± 1.44^a, b^
	250 μmol/L		29.30 ± 1.63^a^	23.70 ± 1.24^a, b^
	500 μmol/L		40.84 ± 2.11^b^	31.98 ± 1.89^a^
	1000 μmol/L		30.20 ± 1.72^a^	21.92 ± 1.35^b^

Different letters (^a, b^) indicate significant differences (*P* < 0.05) between treatments within a given time point for each parameter

**Table 3:** Sperm quality parameters from samples exposed to extender alone (control), or in the presence of phloretin (PHL) at three different concentrations (250 μmol/L, 500 μmol/L and 1000 μmol/L). The evaluated parameters were: percentage of progressively motile spermatozoa (PMOT), percentage of spermatozoa with low membrane lipid disorder (%M540^−^/YO-PRO-1^−^ sperm cells) and percentage of spermatozoa with high mitochondrial membrane potential (MMP; %JC1_agg_ spermatozoa). Determinations were performed in fresh (extended) and frozen-thawed (FT) spermatozoa after 30 and 240 min of thawing. Data reported as mean ± SEM

Variable	PHL concentration	Time point		
		Extended	FT-30 min	FT-240 min
%PMOT	CNT	69.87 ± 3.20	23.38 ± 2.74^a^	10.96 ± 1.12^a^
	250 μmol/L		20.52 ± 0.98^a^	8.61 ± 0.93^a^
	500 μmol/L		22.16 ± 0.75^a^	7.62 ± 0.89^a^
	1000 μmol/L		10.38 ± 0.90^b^	3.82 ± 0.92^b^
%M540^−^/ YO-PRO-1^−^	CNT	80.83 ± 2.79	39.03 ± 1.49^a, b^	31.25 ± 1.06^a^
	250 μmol/L		44.53 ± 1.99^a^	34.19 ± 1.29^a^
	500 μmol/L		37.92 ± 1.37^b^	30.20 ± 1.10^a^
	1000 μmol/L		31.01 ± 1.60^c^	21.42 ± 1.05^b^
%JC1_agg_	CNT	82.79 ± 2.41	31.95 ± 1.89^a^	26.01 ± 1.44^a^
	250 μmol/L		38.21 ± 2.08^a^	22.49 ± 1.21^a^
	500 μmol/L		34.98 ± 2.09^a^	29.29 ± 1.74^a^
	1000 μmol/L		31.93 ± 1.88^a^	25.18 ± 1.29^a^

Different letters (a–c) indicate significant differences (*P* < 0.05) between treatments within a given time point for each parameter

### Effects of PDO on sperm quality

Figure 1 and Table 1 show sperm quality parameters before and after freeze-thawing and the effects of PDO-mediated AQP inhibition during cryopreservation.

Regardless of its concentration, PDO induced a decrease in total sperm motility (*P* < 0.05) at 240 min post-thaw (Fig. 1a), but there were no differences (*P* > 0.05) between the treatments and the control in terms of progressive sperm motility at any post-thaw time point (Table 1). At 30 min post-thaw, the sperm viability (percentage of SYBR-14^+^/PI^−^ spermatozoa) of all PDO treatments was higher than in control samples (*P* < 0.05), but at 240 min post-thaw this difference only persisted in the 1 mmol/L treatment (Fig. 1b). Whereas the percentage of viable sperm with low membrane lipid disorder (percentage of M540^−^/YO-PRO-1^−^ spermatozoa) was significantly (*P* < 0.05) higher than in the control at 30 min post-thaw in the presence of all PDO concentrations, only the 0.1 mmol/L and 1 mmol/L PDO concentrations elicited this effect at 240 min post-thaw (*P* < 0.05; Table 1).

Moreover, addition of 0.1 mmol/L and 1 mmol/L PDO led to a higher MMP (percentage of JC-1_agg_^+^ spermatozoa) with respect to the other treatments and the control at both time points after thawing (*P* < 0.05; Table 1).

The percentage of viable spermatozoa showing high levels of O_2_^−^• (percentage of E^+^/YO-PRO-1^−^ spermatozoa) was higher in PDO-supplemented samples than the control, at both 30 and 240 min post-thaw (*P* < 0.05; Fig. 1c). Similarly, samples treated with different concentrations of PDO presented a higher percentage of viable sperm cells with high peroxide levels (percentage of DCF^+^/PI^−^ spermatozoa) than the control at both 30 and 240 min post-thaw (*P* < 0.05; Fig. 1d).

### Effects of AC on sperm quality

Figure 2 and Table 2 show sperm quality parameters before and after freeze-thawing and the effects of AC-mediated AQP inhibition during cryopreservation.

Neither total nor progressive sperm motility differed between treatments containing AC and the control (*P* > 0.05) (Fig. 2a; Table 2). Regarding viability, it decreased when sperm were exposed to 250 μmol/L AC at 240 min post-thaw (*P* < 0.05; Fig. 2b). Treatment of sperm with AC did not induce changes (*P* > 0.05) in membrane lipid disorder in any of the studied time points compared to the control (Table 2).

In the treatment containing 500 μmol/L AC, the percentage of sperm cells with high MMP was higher than in the control at 30 min post-thaw (*P* < 0.05; Table 2).

In addition, 250 and 1000 μmol/L AC induced an increase in the percentage of viable sperm cells with high O_2_^−^• levels at 30 min post-thaw (*P* < 0.05), but this difference only persisted for the 1000 μmol/L at 240 min post-thaw (*P* < 0.05; Fig. 2c). In contrast, AC had no effect on the percentage of viable spermatozoa with high peroxide levels (Fig. 2d).

### Effects of PHL on sperm quality

Figure 3 and Table 3 show sperm quality parameters before and after freeze-thawing and the effects of PHL-mediated AQP inhibition during cryopreservation.

Concerning PHL, 500 μmol/L concentration reduced total sperm motility at 240 min post-thaw (Fig. 3a; *P* < 0.05). Moreover, PHL at 1000 μmol/L caused a significant (*P* < 0.05) decrease in both total and progressive sperm motilities at any time point after thaw (Fig. 3a, Table 3). Similarly, 1000 μmol/L PHL lowered both sperm viability at 240 min post-thaw (*P* < 0.05; Fig. 3b) and the percentage of viable spermatozoa with low membrane lipid disorder at any time point after thaw (*P* < 0.05; Table 3).

With regard to MMP, no significant differences (*P* > 0.05) were observed in this sperm parameter between treatments and the control (Table 3).

Finally, PHL had no effect either on the percentage of viable spermatozoa with high superoxide levels or on the percentage of viable spermatozoa with high peroxide levels (*P* > 0.05; Fig. 3c, d).

## Discussion

Despite studies conducted in the last decade identifying the presence of AQPs in sperm cells of several mammalian species (reviewed in [17]), neither their precise function nor their mechanism of action have been fully addressed. To this end, this study has used different AQP inhibitors to unveil the relevance of orthodox AQPs and GLPs during cryopreservation. Three different inhibitors were added at three concentrations each: 1,3-propanediol (PDO; 0.1, 1 and 10 mmol/L), acetazolamide (AC; 250, 500 and 1000 μmol/L) and phloretin (PHL; 250, 500 and 1000 μmol/L). PDO has been proven to inhibit orthodox AQPs (AQP1, AQP2, AQP5 and AQP4) with high efficiency, and GLPs (the family to which AQP3, AQP7 and AQP9 belong) with low intensity [20, 21]. With regard to AC, it inhibits AQP1 and AQP4 [22, 23], whereas PHL inhibits both AQP3 and AQP7 [33–35]. The concentrations were based on preliminary experiments conducted to determine the minimum concentration at which an effect was observed, and the maximum concentration at which cytotoxic effects appeared. The assessment of how each inhibitor affected sperm function and survival during cryopreservation was made on the basis of sperm motility, sperm viability, membrane lipid disorder, MMP, and intracellular levels of superoxide and peroxides.

Compared to the control, PDO tended to maintain better post-thaw sperm viability and lower levels of membrane lipid disorder; however, total sperm motility decreased, whereas percentages of spermatozoa with high MMP and with high levels of ROS (including superoxides and peroxides) increased at some concentrations. As far as AC is concerned, there was a lack of consistent effects, and only the percentage of spermatozoa with high MMP and intracellular levels of O_2_^−^• changed at some concentrations and time points. Finally, PHL had detrimental effects on total and progressive sperm motilities, viability and membrane lipid disorder.

The different effects observed between inhibitors might be due to their specificity for different AQPs and the collateral effects on other proteins that are present in the sperm cell. In this context, PDO inhibits a great number of AQPs, especially orthodox AQPs, and, with less efficiency, GLPs. Whereas PDO remains inside the pore of orthodox AQPs because of its narrow diameter, it is able to freely permeate through the GLP pore [20, 21]. Relate to this, it is worth mentioning that Cooper et al. [36] assessed the ability of PDO to penetrate epididymal murine sperm. PDO may disrupt the transport of water and also that of small solutes, including glycerol. Since glycerol is present in many cryopreservation extenders, including the one used in this study, it is possible that its entry to sperm cells and, as a consequence, its function as a CPA, are partially impaired through GLP inhibition. Nevertheless, this hypothesis would not explain why post-thaw sperm viability and the percentage of spermatozoa with low membrane lipid disorder were higher in those samples containing PDO. In this context, one could suggest that PDO could mitigate the negative effects of the lower intracellular concentration of glycerol inside the sperm cell by acting as a CPA itself. In addition, it is widely known that glycerol, much like other CPAs, presents certain toxic effects for sperm cells (reviewed in [15]), and reducing its intracellular concentration could decrease its toxicity. Considering the aforementioned, our results could be explained by PDO lowering the toxic effect of glycerol, and acting as a CPA itself. Consequently, PDO should be further studied as a potential CPA in combination with other agents, as its positive effects on frozen-thawed sperm warrant further research and its impact on sperm viability observed in the current work is coincident with that observed by Widiasih et al. [37] in human sperm. Moreover, PDO has also been used in cryopreservation protocols for canine ovarian cortices [38] and in human multipotent stromal cells [39].

On the other hand, the increase of MMP and ROS levels in PDO-treatments is noteworthy. To the best of our knowledge, the potential effects of PDO on oxidative stress have not been previously reported. Our results suggest that the increase in sperm viability observed when this inhibitor is present in the cryopreservation medium is at the expense of an increase in the percentage of sperm cells with high levels of ROS, and therefore, with potential DNA damage [40]. It is worth mentioning that some studies have unraveled the fact that AQP3 and AQP9 are permeable to H_2_O_2_ [41, 42], and that H_2_O_2_ transport through these proteins does play a vital role in human sperm function [9]. Since the increase of MMP causes an increase in ROS production, including H_2_O_2_, one could suggest that the inhibition of AQP3 and AQP9 by PDO would block H_2_O_2_-efflux. That being said, the aforementioned increase in MMP does not have an apparent cause. A possible explanation could be an interaction of PDO with AQP11, which localizes in the mitochondrial membrane of boar sperm [3]. However, since the inhibiting capacity of PDO with regard to superAQPs has not been previously tested, further studies are needed to unravel the mechanism behind the PDO-mediated increase in MMP.

Regarding the inhibitory mechanism of PHL, it permeates the sperm plasma membrane thanks to its hydrophobic nature [43], and it inhibits GLPs through an internal binding site [44]. Considering its proven inhibitory effect on GLPs, detrimental effects on sperm quality in its presence could be a consequence of the decrease in glycerol permeability, which is consistent with the effects observed in the presence of PDO. In fact, total sperm motility is similarly affected by both inhibitors. Moreover, the detrimental effects on sperm viability and membrane lipid disorder support the hypothesis that PDO rather than PHL exerts a cryoprotective effect. Nevertheless, it is worth highlighting that PHL has inhibitory effects on other sperm proteins, such as SLC2A2 (also known as GLUT2), protein kinase C (PKC), and volume-sensitive and cAMP-activated Cl^−^ channels [45]. All of these proteins can play an important role in the regulation of sperm function: SLC2A2 is involved in the uptake of monosaccharides, which are the main energy source for sperm cells [46]; PKC is implicated in the regulation of sperm motility through phosphorylation of other sperm proteins; and chloride-dependent transport mechanisms are relevant for different sperm cell pathways, such as cAMP-protein kinase A (PKA), which plays a vital role during sperm capacitation [16]. Therefore, not only could PHL mediate the decrease in sperm motility, viability and MMP through the inhibition of GLPs, but also through the inhibition of other sperm proteins.

Concerning AC and its specificity for orthodox AQPs, one would expect that this inhibitor would not affect CPA transport, which could explain why sperm viability and membrane lipid disorder were not affected. Moreover, the fact that AC inhibits AQP1 and AQP4, which have not been previously identified in boar spermatozoa, supports the absence of effects when this inhibitor is added before cryopreservation. In spite of this, some sperm parameters were altered in the presence of AC. While this impact did not appear to depend on the AC concentration, we suggest that it is not directly related to AQP inhibition. Indeed, AC is known to inhibit carbonic anhydrase (CA), and therefore it blocks the conversion of CO_2_ and H_2_O into bicarbonate and protons [47]. Since bicarbonate together with Ca^2+^ stimulates PKA, and complex IV of the electron transport chain is activated via a PKA-mediated phosphorylation, inhibition of CA through AC could cause uncoupling of the electron transport chain [48]. This uncoupling is likely to be responsible for the increase in percentages of spermatozoa with high MMP and with high superoxide levels in AC treatments. Nevertheless, while the intermediate concentration of AC causes an increase in MMP, the highest one has no effect in this parameter compared to the control. Even if no significant differences were found in sperm viability when AC treatments and the control were compared, samples treated with the intermediate concentration of AC showed a significantly (*P* < 0.05) higher viability than those treated with the highest concentration of AC at 30 min post-thaw. Therefore, the absence of differences in MMP in the presence of the highest concentration of AC could be a consequence of mitochondrial alterations that would compromise the integrity of this organelle. In this case, AC would have a hormetic effect on MMP: namely, MMP would have a biphasic response to AC, which would be characterized by a response effect consisting of an increase at low doses, and a decrease at the high ones. Finally, the lack of a concomitant increase in H_2_O_2_ levels might be explained by the normal efflux of this molecule, since rather than targeting GLPs, which present oxygen peroxide permeability, AC inhibit orthodox AQPs only.

## Conclusion

In conclusion, AQP-inhibition effects are highly reliant upon the specificity of the inhibitor and its ability to affect other sperm proteins. In this work, PDO has been found to improve post-thaw sperm survival at the expense of an increase in the percentages of viable spermatozoa with high ROS levels, which suggests its potential role as a CPA. Further research is, however, required to confirm this hypothesis. Based on the observed effects after AC supplementation, which mainly targets orthodox AQPs, it seems that orthodox AQPs are not involved in the response to osmolality variations during freeze-thawing of boar sperm, since the observed changes compared to the control seem to result from collateral effects of this inhibitor upon the sperm cell. Finally, the dramatic impairment observed in post-thaw sperm quality parameters when PHL was added to the cryopreservation media supports the crucial role of GLPs – and not of orthodox AQPs – during boar sperm cryopreservation.

## Data Availability

The datasets and/or analyzed during the current study are available from the corresponding author on reasonable request.

## Abbreviations

AC: Acetazolamide
ALH: Amplitude of lateral head displacement
AQP: Aquaporin
BCF: Beat cross frequency
BTS: Beltsville Thawing Solution
CA: Carbonic anhydrase
cAMP: cyclic AMP
CASA: Computer-assisted sperm analysis
CPA: Cryoprotective agent
DCF^+^: 2′,7′-dichlorofluorescein
E^+^: Ethidium
EV: Electronic volume
FS: Forward scatter
GLP: Aquaglyceroporins
H_2_DCFDA: 2′,7′-dichlorodihydrofluorescein diacetate
H_2_O_2_: Hydrogen peroxide
HE: Hydroethidine
ISAC: International Society for Advancement of Cytometry
JC-1: 5,5′,6,6′-tetrachloro-1,1′,3,3′tetraethyl-benzimidazolylcarbocyanine iodide
JC-1_agg_: JC-1 aggregates
JC-1_mon_: JC-1 monomers
LEY: β-Lactose -Egg yolk -Glycerol
LEYGO: β-Lactose -Egg yolk -Glycerol -Orvus Es Paste
LIN: Linearity
M540: Merocyanine 540
MMP: Mitochondrial membrane potential
O_2_^−^•: Superoxide
PDO: 1,3-propanediol
PfAQP: *Plasmodium falciparum* aquaporin
PHL: Phloretin
PI: Propidium iodide
PKA: cAMP dependent protein kinase
PKC: Protein kinase C
PMOT: Progressive motility
ROS: Reactive oxygen species
SEM: Standard error of the mean
SLC2A2: Solute carrier family 2, facilitated glucose transporter member 2
SS: Side scatter
STR: Straightness
TMOT: Total motility
VAP: Average path velocity
VCL: Curvilinear velocity
VSL: Straight line velocity
WOB: Motility parameter wobble
